# The Impact of a Place-Tailored Digital Health App Promoting Exercise Classes on African American Women’s Physical Activity and Obesity: Simulation Study

**DOI:** 10.2196/30581

**Published:** 2022-08-22

**Authors:** Tiffany M Powell-Wiley, Marie F Martinez, Kosuke Tamura, Sam J Neally, Kelly J O'Shea, Kaveri Curlin, Yardley Albarracin, Nithya P Vijayakumar, Matthew Morgan, Erika Ortiz-Chaparro, Sarah M Bartsch, Foster Osei Baah, Patrick T Wedlock, Lola R Ortiz-Whittingham, Sheryl Scannell, Kameswari A Potharaju, Samuel Randall, Mario Solano Gonzales, Molly Domino, Kushi Ranganath, Daniel Hertenstein, Rafay Syed, Colleen Weatherwax, Bruce Y Lee

**Affiliations:** 1 Social Determinants of Obesity and Cardiovascular Risk Laboratory Cardiovascular Branch, Division of Intramural Research, National Heart, Lung, and Blood Institute National Institutes of Health Bethesda, MD United States; 2 Intramural Research Program National Institute on Minority Health and Health Disparities National Institutes of Health Bethesda, MD United States; 3 Public Health Informatics, Computational, and Operations Research CUNY Graduate School of Public Health and Health Policy New York, NY United States; 4 Center for Advanced Technology and Communication in Health CUNY Graduate School of Public Health and Health Policy New York, NY United States; 5 Socio-Spatial Determinants of Health (SSDH) Laboratory Population and Community Sciences Branch, Intramural Research Program, National Institute on Minority Health and Health Disparities National Institutes of Health Bethesda, MD United States

**Keywords:** computational modeling, digital health, physical activity, BMI, obesity, built environment, impact, app, exercise, simulation, intervention, women, African American, agent

## Abstract

**Background:**

The increasing prevalence of smartphone apps to help people find different services raises the question of whether apps to help people find physical activity (PA) locations would help better prevent and control having overweight or obesity.

**Objective:**

The aim of this paper is to determine and quantify the potential impact of a digital health intervention for African American women prior to allocating financial resources toward implementation.

**Methods:**

We developed our Virtual Population Obesity Prevention, agent-based model of Washington, DC, to simulate the impact of a place-tailored digital health app that provides information about free recreation center classes on PA, BMI, and overweight and obesity prevalence among African American women.

**Results:**

When the app is introduced at the beginning of the simulation, with app engagement at 25% (eg, 25% [41,839/167,356] of women aware of the app; 25% [10,460/41,839] of those aware downloading the app; and 25% [2615/10,460] of those who download it receiving regular push notifications), and a 25% (25/100) baseline probability to exercise (eg, without the app), there are no statistically significant increases in PA levels or decreases in BMI or obesity prevalence over 5 years across the population. When 50% (83,678/167,356) of women are aware of the app; 58.23% (48,725/83,678) of those who are aware download it; and 55% (26,799/48,725) of those who download it receive regular push notifications, in line with existing studies on app usage, introducing the app on average increases PA and decreases weight or obesity prevalence, though the changes are not statistically significant. When app engagement increased to 75% (125,517/167,356) of women who were aware, 75% (94,138/125,517) of those who were aware downloading it, and 75% (70,603/94,138) of those who downloaded it opting into the app’s push notifications, there were statistically significant changes in PA participation, minutes of PA and obesity prevalence.

**Conclusions:**

Our study shows that a digital health app that helps identify recreation center classes does not result in substantive population-wide health effects at lower levels of app engagement. For the app to result in statistically significant increases in PA and reductions in obesity prevalence over 5 years, there needs to be at least 75% (125,517/167,356) of women aware of the app, 75% (94,138/125,517) of those aware of the app download it, and 75% (70,603/94,138) of those who download it opt into push notifications. Nevertheless, the app cannot fully overcome lack of access to recreation centers; therefore, public health administrators as well as parks and recreation agencies might consider incorporating this type of technology into multilevel interventions that also target the built environment and other social determinants of health.

## Introduction

The increasing prevalence of smartphone apps to help people find different services (eg, Yelp and OpenTable to find restaurants, Fandango to find movie theaters, AllTrails to find hikes, GasBuddy to find gas stations, Expedia to find hotels, and Zillow to find homes and apartments) raises the question of whether apps to help people find physical activity (PA) locations (eg, ClassPass [1] and Fit Reserve [2]) would help to better prevent and control having overweight and obesity. Such place-tailored apps can help assemble, collate, and present information that may be available on different websites so that an individual can quickly find the closest location of interest. These place-tailored apps can be particularly helpful for PA locations and opportunities since they may exist in different and less obvious forms (eg, irregular timing of classes, walking and bike paths, outdoor tracks, and tennis or basketball courts). Such an app can also offer crowdsourced ratings of each location, details about specific services (eg, time, availability, costs, promotions, and deals), and even social connections with people who have the same interests or are in the same area. Previous studies have shown that people may not be aware of or have difficulty finding locations to engage in PA [3-5]. This may be the case in underresourced and otherwise disadvantaged communities where parks, affordable gyms, and other opportunities may be more difficult to find if they are in less-frequented or obscure locations, or if they are not regularly advertised or promoted [6]. African American women who live disproportionately in underresourced communities spend at least as much time as any other racial or ethnic group using apps and the internet (approximately 19 hours and 27 minutes each week versus 17 hours and 8 minutes each week), and approximately 80% of African American women own a smartphone [7], raising the possibility that this could be an effective means to help these women find PA opportunities. However, before such an app is rolled out in the “real world,” it can be helpful to use simulation modeling to guide the design and test the potential impact of such an app. Such an approach is used in other fields (eg, aeronautical engineering and manufacturing) since running simulation models can take much less time and can be significantly less costly than conducting a real-world trial (which can take months to set up, recruit for, and implement). Moreover, once a trial is completed, one cannot go back and change the circumstances as they can in a simulation model. Therefore, we further developed our agent-based simulation model of Washington, DC to test the impact of such a place-tailored digital health app.

## Methods

### Ethics Approval

All authors’ institutions were included in the institutional review board approval (IRB #00004203) at Johns Hopkins as the study began while certain members of the research team (MCF, KJO, YA, MM, SMB, PTW, SS, SR, MSG, MD, KR, DH, RS, and BYL) were based at Johns Hopkins.

### Model of Washington, DC

We used and further developed a Virtual Population Obesity Prevention, agent-based model of Washington, DC in 2020-2021 [8,9], which includes computer model–based representations of households, workplaces, and recreation centers throughout all 8 wards (similar to districts in other cities) in Washington, DC.

### Agents Representing People

We represented each of the 167,356 African American women (aged 18-65 years) living in Washington, DC with a computer model–based agent. Each agent (ie, each African American woman in Washington, DC) has attributes for age, height, lean or fat mass, household location, work location, and income based on representative data for the region and population. Each agent also has an embedded metabolic model, which converts daily caloric intake and expenditure to corresponding lean or fat mass [10,11]. Caloric expenditure from exercise is determined by exercise intensity, duration, and the agent’s current body weight [10,11]. Since individuals may vary in their inclination to exercise, each agent had a baseline probability of wanting to exercise each day. This accounts for an agent’s past experiences and existing tendencies to exercise and includes factors such as household financial status, family responsibilities, chronic health conditions, and social influences. Different scenarios ranged this baseline probability from 10% (10/100) to 50% (50/100) to explore how this probability might affect the results.

**Figure 1:**
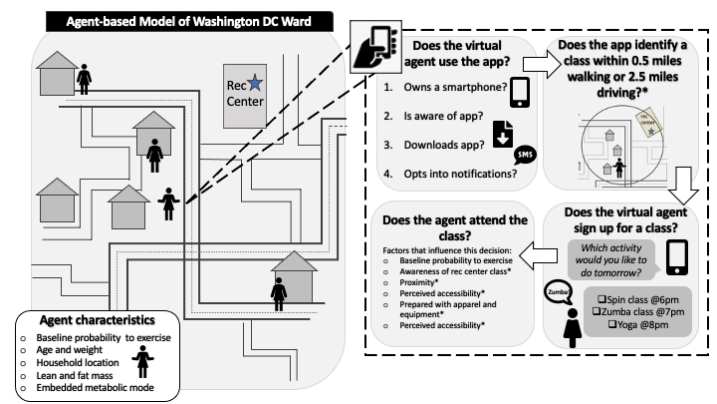
A digital health app that helps locate and send reminders about recreation (rec) center classes. *Factors influenced by phone app.

In each simulated day, women may participate in a recreation center class, depending on a number of factors (Figure 1; Multimedia Appendix 1, Table S1 [8, 9, 12-23]), including the following: (1) her baseline probability to exercise (this accounts for an agent’s past experience and existing tendencies to participate in recreation center classes), which we vary between simulation experiments; (2) objective accessibility to locations, based on the geographic locations of recreation centers [12], the distance individuals need to travel to reach these locations, and access to the types of transportation (eg, cars) that might be required to reach locations further away [24,25]; (3) perceived accessibility of locations [15], based on the individual’s understanding and knowledge of nearby recreation centers; (4) awareness of classes at recreation centers; and (5) preparedness to exercise (whether or not she remembers her apparel and equipment).

### Representations of Recreation Centers

Multimedia Appendix 1, Table S2 shows key characteristics (eg, number of recreation centers) for each ward. If an agent ultimately participates in a recreation center class, she is active for 50 minutes [12] at an intensity of approximately 6.5 metabolic equivalents [16].

### Representations of Digital Health App

In the model, we represent a digital health app that helps locate and send reminders about in-person recreation center classes to increase the agents’ likelihood of participation (Figure 1). This mobile app uses a database of public locations that have been previously identified as locations for PA, such as recreation centers in the case of this paper. Once this registry is verified, a geofence—a geographic boundary—can be created within the mobile app with a set distance surrounding the chosen location; in this case, our simulated app searched for recreation centers within 0.5 miles of the user. When the simulated mobile app detects that the user is within this defined boundary, it will generate a notification that will alert the user of the available resources in the area. Unlike existing fitness apps, this digital health app considers the geographic location of the user and the recreation centers to connect agents with recreation center classes that align with their neighborhood environment and schedules; prompts users to remind them about upcoming classes and what equipment they will need; and provides individually tailored information about class time, location, and necessary equipment to maximize user engagement (Figure 1). If an agent has a smartphone, downloads the app, and opts into notifications (Multimedia Appendix 1, Table S1), the app will send a question each evening asking the user which activity or class she would like to participate in the following day, thereby increasing an agent’s knowledge of class schedules. After selecting the class, agents will receive a notification with a reminder of the class’s time, location, and activity, as well as a reminder to bring clothes or equipment, thereby increasing an agent’s probability of being prepared for and attending class. When representing the digital health app, we introduced it at the beginning of the 5-year simulation, but not all participants continued to use the app for the entire simulation duration (eg, we represented attrition, people discontinuing app use, during the 3 months following the introduction of the app; Multimedia Appendix 1, Table S1).

### Representations of Engagement With the Digital Health App

Since only a certain percentage of the population may be aware that the app is available, we varied the proportion of women across the population who, in a given scenario, were aware of the app, subsequently downloaded the app, and then opted into push notifications (25%-75%). This means, 25% (41,839/167,356) of women are aware of the app, 25% (10,460/41,841) of those who are aware download it, and 25% (2615/10,460) of those who download it receive regular push notifications from the app. We ranged this to 75% (125,517/167,356) of women aware of the app, 75% (94,138/125,517) of those who are aware download it, and 75% (70,603/94,138) of those who download it opt into the app’s push notifications. Varying the level of user engagement across a range can help identify the thresholds of app engagement that result in observable and statistically significant impacts on PA and weight.

### Simulation Experiments

We used the model of Washington, DC to simulate the impact of a digital health app on in-person recreation center class participation, recreation center class PA (minutes per week), subsequent changes in BMI, as well as the prevalence of obesity and the state of having overweight. Each simulation experiment consisted of running the model of Washington, DC and all 167,356 computer model–based agents, 10 times over 5 simulated years.

### Validation

Validation consisted of comparing different model-generated metrics to observed values to determine if the model was representing what was occurring. For example, when we ran simulation runs, we saw that, on average, 2.1% (3514/167,356) of women were participating in recreation center classes daily compared to the observed 3.8% from the 2017 American Time Use Survey [17]. Since the people who exercised on one day will not necessarily be the same people who exercised on a different day, there will be a certain proportion of the population that exercised at least once over the course of the month. Thus, we also simulated the average percentage of women participating in recreation center classes at least once on a monthly basis (19.1% [31,965/176,356]) and compared this to the observed proportion of women participating in workout class activity on a monthly basis (16.1%), as reported by the Behavioral Risk Factors Surveillance System [18]. The model-generated data generally matched the observed data, and the differences are likely due to differences between populations and the classes available to that population. Further model validation details are available in Multimedia Appendix 1.

## Results

### No Mobile App

**Figure 2:**
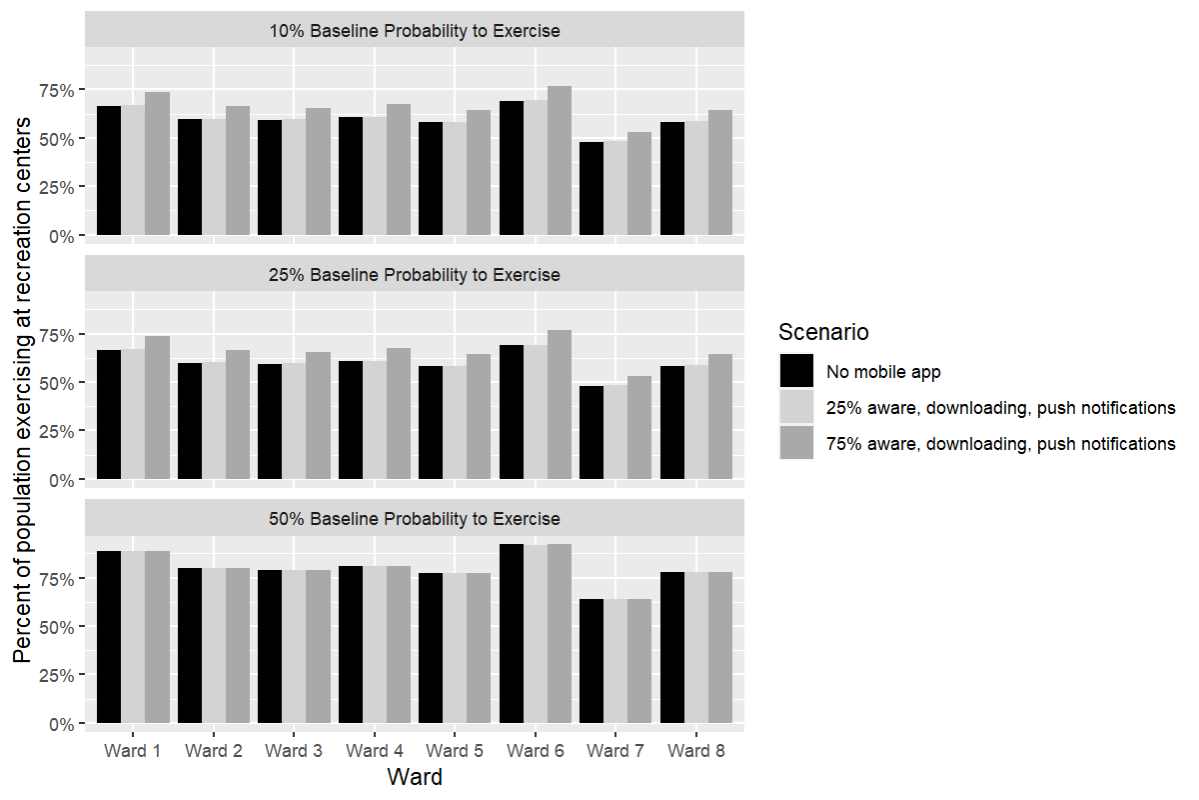
Percent of women exercising with and without the mobile app within each ward in Washington, DC.

Table 1 shows PA from recreation center classes and weight-related outcomes after 5 years with no mobile app for different baseline probabilities to exercise. Figure 2 shows how the percent of women who exercised at least once in the simulation when there was no app varied by the Washington, DC ward. For example, Ward 6 had the highest percent of the population who exercised at least once (69.1% [4331/20,739], 95% CI 68.9%-69.2%), while Ward 7 had the lowest (48% [15,710/32,729], 95% CI 47.9%-48.0%) when there was no mobile app (25% [25/100] baseline probability to exercise). This trend in ward-level variation was consistent across all baseline exercise probabilities.

**Table 1:** Physical activity, overweight, obesity, BMI outcomes by baseline probability to exercise for different scenarios (eg, with and without digital health app).

Simulation scenarios at each baseline probability to exercise				Percent of population exercising at recreation centers, mean (95% CI)		Average number of physical activity min/week, mean (95% CI)		Overweight prevalence, mean (95% CI)		Obesity prevalence, mean (95% CI)		Average BMI, mean (95% CI)		Average BMI among women with obesity, mean (95% CI)
**10% (10/100) baseline probability to exercise**														
	No digital health app		58.66 (54.65-62.67)		36.97 (34.45-39.50)		24.44 (23.91-24.97)		56.10 (54.56-57.64)		30.16 (29.86-30.45)		34.20 (34.00-34.41)	
	**Introducing place-tailored digital health app**													
		25%-25%-25%^a^	58.91 (54.87-62.94)		37.26 (34.71-39.81)		24.42 (23.88-24.96)		56.09 (54.53-57.65)		30.15 (29.86-30.45)		34.21 (34.00-34.43)	
		50%-50%-50%^b^	61.09 (56.92-65.26)		39.83 (37.12-42.54)		24.45 (23.91-24.98)		55.67 (54.15-57.19)		30.07 (29.78-30.36)		34.16 (33.94-34.37)	
		75%-75%-75%^c^	65.10 (60.64-69.56)		44.45 (41.41-47.50)		24.70 (24.21-25.20)		54.68 (53.12-56.25)		29.90 (29.60-30.19)		34.04 (33.83-34.26)	
**25% (25/100) baseline probability to exercise**														
	No digital health app		58.67 (54.66-62.68)		52.84 (49.23-56.45)		25.52 (25.04-26.01)		52.75 (51.06-54.43)		29.56 (29.27-29.86)		33.81 (33.61-34.01)	
	**Introducing place-tailored digital health app**													
		25%-25%-25%^a^	58.92 (54.89-62.94)		53.25 (49.61-56.89)		25.54 (25.04-26.04)		52.62 (50.91-54.33)		29.56 (29.26-29.86)		33.83 (33.62-34.05)	
		50%-50%-50%^b^	61.17 (56.99-65.35)		56.98 (53.09-60.88)		26.24 (25.68-26.80)		51.25 (49.47-53.04)		29.44 (29.14-29.74)		33.83 (33.62-34.03)	
		75%-75%-75%^c^	65.10 (60.64-69.55)		63.52 (59.18-67.87)		27.72 (27.05-28.40)		48.66 (46.75-50.56)		29.23 (28.92-29.54)		33.82 (33.63-34.02)	
**50% (50/100) baseline probability to exercise**														
	No digital health app		78.30 (72.96-83.64)		86.33 (80.43-92.22)		27.88 (26.36-29.39)		44.42 (41.63-47.20)		28.38 (28.06-28.70)		33.00 (32.80-33.21)	
	**Introducing place-tailored digital health app**													
		25%-25%-25%^a^	78.22 (72.86-83.58)		86.88 (80.93-92.83)		28.24 (26.81-29.67)		43.90 (41.27-46.52)		28.38 (28.05-28.71)		33.08 (32.84-33.31)	
		50%-50%-50%^b^	78.25 (72.90-83.60)		92.17 (85.89-98.45)		28.57 (27.17-29.96)		42.63 (40.03-45.23)		28.24 (27.91-28.57)		33.10 (32.86-33.34)	
		75%-75%-75%^c^	78.29 (72.95-83.63)		101.41 (94.48-108.33)		29.40 (28.15-30.66)		40.27 (37.75-42.78)		28.00 (27.65-28.34)		33.15 (32.91-33.40)	

^a^25% (41,839/167,356) aware of the app, 25% (10,460/41,839) of those who are aware download the app, and 25% (2615/10,460) of those who download it receive notifications.

^b^50% (83,678/167,356) aware of the app, 50% (48,725/83,678) of those who are aware download the app, and 50% (26,799/48,725) of those who download it receive notifications.

^c^75% (125,517/167,356) aware of the app, 75% (94,138/125,517) of those who are aware download app, and 75% (70,603/94,138) of those who download it receive app notifications.

### Impact of Introducing a Place-Tailored Mobile App That Connects Users to Recreation Center Classes

With lower levels of user engagement with the mobile phone app, that is 25% aware of app (41,839/167,356), 25% of those aware download app (10,460/41,839), and 25% (2615/10,460) of those who download it receive app notifications, the app had a negligible and nonsignificant impact on the additional minutes of PA (<1 minute), on the additional percent of women who ever exercise (0.2% [335/167,356]; Figure 2), and on reductions in obesity prevalence (0.1% [167/167,356]). Thus, even lower levels of app engagement (eg, below 25% [25/100]) would have no effect on physical activity and weight.

**Figure 3:**
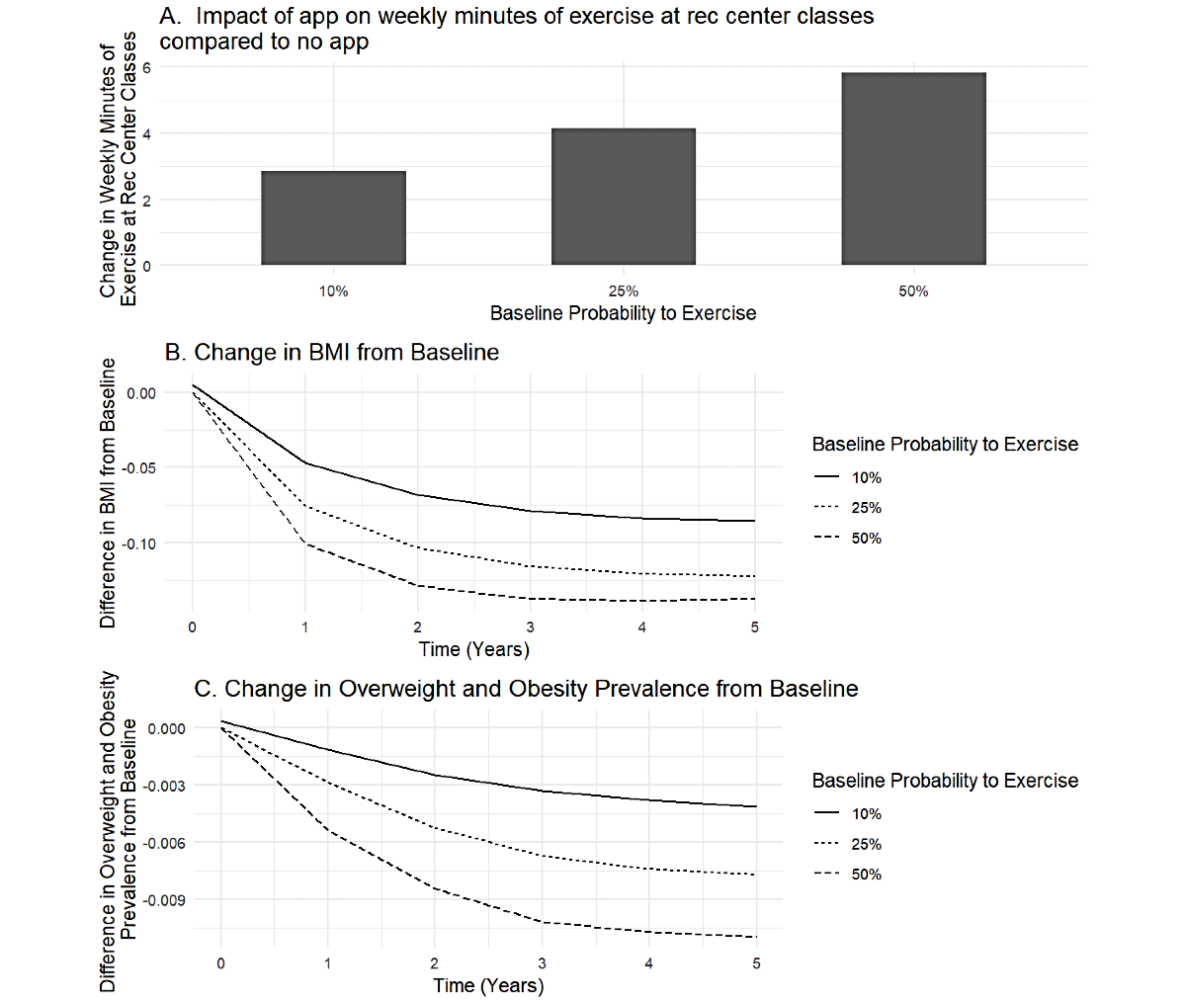
Impact of mobile app on physical activity, BMI, as well as overweight and obesity prevalence at each baseline probability to exercise. Rec: recreation.

Increasing user engagement to approximately 50% (eg, 50% aware [83,678/167,356], 58.23% [48,725/83,678] of those who are aware download the app [19], and 55% [26,799/48,725] of those who download it receive regular push notifications [20]) resulted in moderate improvements to PA from recreation center classes and weight-related outcomes across the population. Figure 3 shows these observable changes to PA (panel A), BMI (panel B), and overweight and obesity prevalence (panel C). With a 10% (10/100) baseline probability of exercise, the PA minutes per week increase by 2.9 minutes (95% CI –1.4 to 17.9), BMI decreases by 0.09 kg/m^2^ (95% CI –0.56 to 0.39), and obesity prevalence decreases by an absolute 0.43% (720/167,356; 95% CI –2.7% to 2.93%) at the end of the 5-year simulation. When baseline probability increases to 50% (50/100), there are larger increases in weekly PA minutes (5.4 minutes, 95% CI –4.1 to 15.8), and larger reductions in BMI (0.14 kg/m^2^, 95% CI –0.67 to –0.4) and obesity prevalence (1.8% [3012/167,356]; 95% CI –2.6% to 6.2%).

The percent of women attending at least one recreation center class over the course of the simulation shows additional gains between when the baseline probability to exercise is between 10% (10/100; 2.43% [4067/167,356], 95% CI –4.24% to 9.1%) and 25% (25/100; 2.5% [4184/167,356]; 95% CI –4.2% to 9.2%). When the baseline probability to exercise is 50% (50/100), the percent of women exercising at least once hits a ceiling of 78% (130,538/167,356) (increase of 0.05% [84/167,356]; 95% CI –8.68% to 8.77%), due to the location and accessibility of recreation centers for some women. Thus, at lower probabilities to exercise (eg, 10%-25%), the app is more effective at increasing the number of women participating in at least one recreation center class (Figure 2). However, additional PA minutes per week from recreation center classes increase with baseline probability to exercise (eg, 4.14, 95% CI –1.9 to 10.2 vs 5.9, 95% CI –4.1 to 15.7 minutes per week at 25% [25/100] and 50% [50/100] baseline probabilities to exercise, respectively; Figure 3). Figure 3 also shows how reductions in BMI and overweight and obesity prevalence due to app use accrue over time during the 5-year simulation.

Further increasing app engagement to 75%, with 75% (125,517/167,356) of women aware of the app, 75% (94,138/125,517) of those who are aware downloading the app, and 75% (70,603/94,138) of those who download it opting into the app’s push notifications resulted in statistically significant gains to PA and reductions in obesity prevalence. For example, weekly PA increased by 10.7 (95% CI 4.2-17.2) minutes per week, and obesity prevalence decreased by an absolute 4.09% (6,845/167,356; 95% CI 1.2%-7.0%) with 25% baseline exercise probability (Table 1).

### Ward-Level Impact of Place-Tailored Mobile App

The results varied substantially by ward. For example, at 25% (25/100) baseline probability to exercise (assuming 50% [83,678/167,356] aware, 50% [48,725/83,678] of those who are aware downloading the app, and 50% [26,799/48,725] of those who download it receiving app notifications), Ward 6 had the highest absolute increase in average PA minutes per week (4.85, 95% CI 4.58-5.11), and the greatest reduction in average BMI (–0.15 kg/m^2^, 95% CI –0.19 to –0.11). However, Ward 7 had the lowest (3.39, 95% CI 3.24-3.53) increase in PA minutes per week and the smallest reduction in BMI (–0.09 kg/m^2^; 95% CI –0.12 to –0.06). Changes in overweight and obesity prevalence also varied between wards and decreased by as much as 2.6% (539/20,739; 95% CI 2.3%-2.9%) in Ward 6, where participation in recreation center classes was highest and as little as 1.9% (622/32,729; 95% CI 1.7%-2.1%) in Ward 7 (25% baseline exercise probability).

## Discussion

### Principal Findings

Our simulation model of African American women in Washington, DC, and their use of a place-tailored digital health app to help identify recreation center classes shows that the app does not result in substantive population-wide health effects at lower levels of app engagement (eg, 25% of women are aware of the app, 25% of those aware of the app download it, and 25% of those who download it receive regular push notifications from the app). When 50% of women are aware of the app, 58.23% of those who are aware download the app, and 55% of those who download it receive regular push notifications from the app, there are observable changes in PA and weight across the population, but the impact is not statistically significant. For the app to result in statistically significant increases in PA and reductions to obesity prevalence over 5 years, there needs to be at least 75% of women who are aware of the app, 75% of those aware of the app downloading it, and 75% of those who download it opting into the app’s push notifications. Thus, we demonstrated the minimum levels of engagement needed at the outset of a mobile phone app campaign (approximately 50% aware of the app, 50% of those who are aware download the app, and 50% of those who download it receive app notifications, assuming reductions in use over the first 3 months) to observe a change in PA and weight across the population. Studies have shown how perceived usefulness of an app, user-friendliness, backing from health care professionals, and continued engagement impact app usage [26,27] could be addressed through a structured marketing and communications strategy. Thus, future interventions should prioritize efforts to increase marketing for the place-tailored app to increase the percent of women who are aware of and use the app to reach the impactful threshold of engagement and obtain further benefits.

Further, our results show that a place-tailored app is more likely to be successful in increasing PA in those who already have a higher likelihood to exercise. While the results showed that the app was successful at encouraging individuals who have a low baseline probability (eg, 10% [10/100] and 25% [25/100]) to exercise to attend at least one new class over the course of the simulated period, this alone was not enough to drive a sustained change in regular exercise. The app did a better job at increasing the average duration of PA each week as baseline probability to exercise increased. This indicates that improving knowledge of recreation center classes, while important, should be coupled with interventions to help overcome personal and social barriers (eg, limited social support for PA or time constraints) that determine baseline exercise probability [28,29]. Place-tailored digital health apps could potentially address some of these barriers through the release of new features and functionality such as a social networking component [30,31].

Regardless of user engagement with the app, place-tailored digital health apps need to be combined with increasing physical access to recreation centers to see greater than additive effects in PA and subsequent health outcomes. There is a limit to a place-tailored app’s impact because some individuals cannot access recreation centers due to the distance and lack of transportation (eg, access to car) from their home location. As shown in our results, there are clear disparities in the success of the app in improving health outcomes in neighborhoods with greater access to recreation centers (with nearly a 1.4-fold increase in the use of recreation center classes in these neighborhoods [eg, Ward 6]) compared to neighborhoods with less accessible recreation centers (eg, Ward 7), even with 75% of women who are aware of the app, 75% of those aware of the app download it, and 75% of those who download it opt into the app’s push notifications. Past studies have shown that lower-income neighborhoods in many cities around the United States have less accessible PA locations and recreation centers [32]. Therefore, it is important for public health administrators and park and recreation agencies to consider pairing this type of digital health technology with improvements to recreation center access such as changes to the built environment, perceived safety, or transportation.

Our results also show that it takes time for the effect of the place-tailored mobile app to fully manifest (>2 years). In general, 1 year is not enough time to see an impact on BMI and overweight and obesity prevalence, as population-level effects on weight and subsequent health benefits accrue over years. This shows the need to continuously measure the value of intervention programs over a period of several years, since reductions in overweight and obesity prevalence may not be demonstrated immediately, and effective interventions may wrongly be deemed unsuccessful if evaluated too early. Accounting for this ramp-up period is important, as it can also take time for a new technology to be adopted and used. Our results show that the speed of the reduction in overweight and obesity prevalence in the population increases year after year as adoption rates increase, revealing a potential opportunity to increase momentum as more users adopt similar place-tailored digital health technology.

In addition to being able to simulate extended periods of time, another benefit of simulation modeling is that it can be adapted and refined over time. For example, simulation modeling can be used in conjunction with clinical trials [33,34] so that the model can continuously inform digital health phone app design and multipronged PA interventions. The simulation model can be run first, to help determine the impact of an app, which can then inform the implementation of a trial. Data and information from the trial can then further update the model. This iterative process can continue until the app or intervention is optimally designed.

### Limitations

All models are simplifications of reality and cannot account for all possible factors that may affect PA decision-making. Our model included a few simplifying assumptions. For example, we did not account for objective accessibility to a recreation center near a woman’s workplace and used the objective accessibility near the home as a proxy. In addition, since we wanted to demonstrate how to design an app that harnesses geographic location and the value of such an app, our study focused on the app locating and reminding individuals about in-person classes, rather than web-based classes. However, such an app may offer similar benefits for web-based classes such as reminding individuals about when classes are scheduled and what equipment is needed, while reducing potential geographic barriers to exercise. We also assumed that in-person classes are available (eg, not during a public health emergency such as the COVID-19 pandemic). When determining body weight changes for each woman, we assumed that compensatory eating did not occur. Our model simulated behavior of and used data specific to Washington, DC African American women, which may limit generalizability to other populations or geographic areas.

### Conclusions

Our study shows that a digital health app that helps identify recreation center classes does not result in substantive population-wide health effects at lower levels of app engagement (eg, 25% of women who are aware of the app, 25% of those who are aware of the app download it, and 25% of those who download it receive regular push notifications from the app). For the app to result in statistically significant increases in PA and reductions to obesity prevalence over 5 years, there needs to be at least 75% of women aware of the app, 75% of those aware of the app download it, and 75% of those who download it opt into the app’s push notifications. Even so, the app cannot fully overcome lack of access to recreation centers, and therefore, public health administrators as well as parks and recreation agencies might consider incorporating this type of technology into multilevel interventions that also target the built environment and other social determinants of health.

## Appendix

Multimedia Appendix 1 Supplementary materials.

## Abbreviations

PA: physical activity
