# Supplementary material for: The Impact of a Place-Tailored Digital Health App Promoting Exercise Classes on African American Women’s Physical Activity and Obesity: Simulation Study
Source: J Med Internet Res. 2022 Aug 22;24(8):e30581. doi: 10.2196/30581 (PMC9446149; doi:10.2196/30581)
Supplement: Multimedia Appendix 1 [file jmir_v24i8e30581_app1.docx]

**Multimedia Appendix 1. Supplementary materials.**

*Model Validation*

We validated model outputs in two different ways. First, we compared the proportion of women participating in recreation center classes in the baseline scenario to real world data. The 2017 American Time Use Survey (ATUS) asks a nationally representative sample of Americans how they spent their time during the previous day, showing that 3.8% of women exercised at a gym or health club the prior day for an average of 58.4 minutes [17]. On an average simulated day (sampled 100 random days), 2.1% of AA women in D.C. exercised at a recreation center; classes lasted 50 minutes each. As respondents’ race cannot be identified in the ATUS, it likely overestimates the percentage of people who reported exercising as the sample includes women who are active at higher average rates than AA women.

Second, we used the Behavioral Risk Factor Surveillance System (BRFSS), a survey that asked AA women in Washington, D.C. about their exercise habits [18]. We created a list of activities that people likely did in a gym or recreation center and BRFSS reports 16.1% of respondents had done one of those activities in the prior 30 days. In our simulations, an average of 19.1% of agents exercised at a recreation center in a given month.

*Data Sources*

As described in previous publications [8,9], our synthetic population integrates data from the U.S. Census Bureau to identify sociodemographic information and residential locations for each agent in the model [13]. Each agent has a starting height and weight based on the 2014 National Health and Nutrition Examination Survey (NHANES). Class durations were based on posted schedules for recreation center classes [14] and class intensity parameters were based on average the metabolic equivalents (METS) listed in the compendium for PA for common recreation center class activities [16]. The probability of car ownership is based on the D.C. ward in which an agent lives [21]. Initial rates of app usage were based on data from the literature and Pew Research Surveys (S1).

Table S1

| Model Input Parameter | Value | | Source |
| --- | --- | --- | --- |
| Percent of people who do not have access to a car | Ward 1 | 41% | [21] |
|  | Ward 2 | 46% |  |
|  | Ward 3 | 21% |  |
|  | Ward 4 | 22% |  |
|  | Ward 5 | 33% |  |
|  | Ward 6 | 35% |  |
|  | Ward 7 | 41% |  |
|  | Ward 8 | 48% |  |
| Awareness of Recreation Center Schedule | 75% | | *Varied with sensitivity analyses* |
| Prepared for exercise with equipment and apparel | 90% | |  |
| Perceived accessibility | 59% | | [15] |
| Length of Recreation Center class | 50 minutes | | [12] |
| Intensity of Recreation center class | 6.5 METS | | [16] |
| Mobile Phone App Parameter | Value | | Source |
| Proportion of population with a smartphone | 77% | | [22] |
| Proportion of population aware of App | 50% | | *Varied with sensitivity analyses* |
| Proportion of population that has downloaded the App | 58.23% | | [19] |
| Proportion of population that receives regular push notifications | 55% | | [20] |
| Proportion of people who continue using app by month | Month 1 | 43% | [23] |
|  | Month 2 | 34% |  |
|  | Month 3 | 29% |  |

TableS2. Washington DC ward-level characteristics

| Ward | Population size (AA women) | Total recreation centers | Mean distance to recreation center (meters) | Standard deviation | Minimum distance to recreation center | | Maximum distance to recreation center | Car ownership (%) |
| --- | --- | --- | --- | --- | --- | --- | --- | --- |
| 1 | 15120 | 7 | 483 | 190 | 68 | 1193 | | 59 |
| 2 | 7476 | 5 | 786 | 506 | 110 | 1838 | | 54 |
| 3 | 5227 | 10 | 863 | 282 | 482 | 1511 | | 79 |
| 4 | 20931 | 11 | 839 | 404 | 35 | 2095 | | 78 |
| 5 | 33804 | 12 | 594 | 271 | 36 | 1591 | | 67 |
| 6 | 20739 | 8 | 495 | 244 | 134 | 1839 | | 65 |
| 7 | 32729 | 10 | 654 | 234 | 88 | 1757 | | 59 |
| 8 | 31330 | 10 | 509 | 279 | 32 | 1517 | | 52 |
